# The serum creatinine to cystatin C ratio predicts the risk of acute exacerbation of chronic obstructive pulmonary disease

**DOI:** 10.1097/MD.0000000000033304

**Published:** 2023-03-24

**Authors:** Liang He, Yan Li, Xijun Gou, Ling Lei

**Affiliations:** a Department of Respiratory and Critical Care Medicine, Xindu District People’s Hospital, Chengdu, Sichuan Province, China; b Department of Intensive Care Medicine, Xindu District People’s Hospital, Chengdu, Sichuan Province, China.

**Keywords:** acute exacerbation of chronic obstructive pulmonary disease, creatinine, cystatin C

## Abstract

The purpose of acute exacerbation of chronic obstructive pulmonary disease (AECOPD) treatment is to minimize the negative impact of the current exacerbation and to prevent the development of subsequent events. Therefore, it is important to identify readily available serological indicators during hospital admission to assess the prognosis of patients with AECOPD. All patients hospitalized in a Department of Respiratory and Critical Care Medicine of tertiary care hospital between January 2021 and December 2021 for AECOPD were analyzed using univariate correlations and binary logistic regression analysis with 2 models for associations between demographic, clinical, and laboratory features and AECOPD risk. The ratio of creatinine to cystatin C (Cre/Cys C) ratio was significantly associated with age (r = −0.206, *P* = .000), weight (*R* = 0.331, *P* = .000), body mass index (BMI) (*R* = 0.133, *P *= .007), and forced vital capacity (FVC)% predicted (*R* = 0.130, *P* = .009). Multiple regression was performed to predict the Cre/Cys C ratio from age, weight, BMI, forced expiratory volume during 1 second/FVC ratio, and FVC% predicted FABP-4, with F (5, 405) = 24.571, *P* = .000, R2 = 0.233. The results showed that the most significant predictors of the Cre/Cys C ratio were age (*P* = .007), weight (*P* = .000), BMI (*P* = .000), and predicted forced expiratory volume during 1 second (*P* = .000). Multivariate analysis was performed to determine whether the Cre/Cys C ratio was a predictor of AECOPD risk. Model 1 showed that a low Cre/Cys C ratio was associated with an increased hospital length of stay (odds ratio: −0.114, 95% confidence interval: −0.061 to −0.005) and admission to the intensive care unit (odds ratio: 0.951, 95% confidence interval: 0.907–0.996). After adjustment for potential confounding factors, model 2 showed that a low Cre/Cys C ratio was not independently associated with AECOPD risk. The present study indicated that the Cre/Cys C ratio is an easy, cheap, repeatable, and promising tool that allows us to evaluate the risk of AECOPD using serum markers. A low Cre/Cys C ratio was associated with a prolonged hospital length of stay and admission to the intensive care unit in AECOPD patients. However, the associations were not independent.

## 1. Introduction

Chronic obstructive pulmonary disease (COPD) is currently the 4th leading cause of death in the world but is projected to be the 3rd leading cause of death by 2020. Many people suffer from this disease for years and die prematurely from it or its complications.^[1]^ COPD has a chronic and progressive course that is often punctuated by “exacerbations,” which are driven by respiratory infections and multiple functional impairments and comorbidities.^[2–4]^ The purposes of acute exacerbation of chronic obstructive pulmonary disease (AECOPD) treatment are to minimize the negative impact of the current exacerbation and to prevent the development of subsequent events.^[5]^ Acute exacerbation of COPD (AECOPD) is a primary cause of hospitalization and is associated with high mortality.^[6]^Therefore, it is important to identify serological indicators that are readily available at the time of hospital admission to evaluate the prognosis of patients with AECOPD.

The ratio of creatinine to cystatin C (Cre/Cys C) was first reported as a surrogate marker of muscle mass in 2013.^[7]^ It is noninvasive, fast, and less expensive than other sarcopenia examination methods, such as magnetic resonance imaging and computed tomography.^[8]^ On this basis, Cre/Cys C has been proven to be an accurate and inexpensive predictor of sarcopenia in patients with COPD,^[8]^ type 2 diabetes,^[9]^ and cancer.^[10]^ Moreover, it is correlated with glucose disposal ability and diabetic complications in patients with type 2 diabetes.^[9]^ Sarcopenia is common in patients with COPD,^[11]^ which may affect both respiratory muscles and limb muscles and could, therefore, have critical consequences related to the quality of life and prognosis of COPD patients.^[11,12]^ However, to date, no studies have evaluated the relationship of Cre/Cys C levels upon admission for AECOPD with the outcome of hospitalization. As it is a routine blood test, a detailed analysis of Cre/Cys C levels relation to risk seems worthwhile. Therefore, this study aimed to investigate the association between Cre/Cys C levels and risk in patients with AECOPD.

## 2. Methods

### 2.1. Study design and patient recruitment

We retrospectively and consecutively enrolled AECOPD admissions to the Department of Respiratory and Critical Care Medicine of Xindu District People’s Hospital between January 2021 and December 2021. Patients who received treatment according to the global initiative for chronic obstructive lung disease (GOLD) were invited to participate. The participants were divided into 2 groups based on their Cre/Cys C ratio values: the low Cre/Cys C ratio group and the high Cre/Cys C ratio group. The median Cre/Cys C ratio was 74.49, which was used as the threshold value, below which the Cre/Cys C ratio value was considered low.^[13]^ The median value and any values above it were considered high Cre/Cys C ratio values.

Criteria for inclusion: The diagnosis of AECOPD was established based on the criteria of the GOLD.^[1]^ Exacerbation of COPD was defined as an acute worsening of respiratory symptoms that resulted in additional therapy. All patients included in the study have completed blood routine and biochemical indicators, and complete clinical characteristic data.

The following patients were excluded: Age <18 years; Other potential causes of sarcopenia (malignant diseases, heart failure, hyperthyroidism, or any other devastating chronic disease); Chronic kidney disease or acute kidney injury; Concomitant active malignancy; and Incomplete clinical records.

The study was carried out in accordance with the Declaration of Helsinki and was approved by the Ethical Review Committee of the Xindu District People’s Hospital of Chengdu. Due to the retrospective nature of the study and the anonymity of the data, informed consent was not required from the patients.

### 2.2. Pulmonary function tests

All the enrolled patients underwent spirometry, and the forced expiratory volume during 1 second (FEV1) and forced vital capacity (FVC) were measured according to the GOLD consensus guidelines.^[1]^ The FEV1, FEV1/FVC, and the ratio of FEV1 to predicted FEV1 after inhaling bronchodilators were recorded. According to the category of airflow limitation in COPD (based on FEV1 after the administration of a bronchodilator) in patients with FEV1/FVC < 0.70, stable COPD was divided into 4 subgroups: mild (GOLD 1), ≥80% of predicted FEV1; moderate (GOLD 2), 50% to 80% of predicted FEV1; severe (GOLD 3), 30% to 50% of predicted FEV1; very severe (GOLD 4), <30% of predicted FEV1.

### 2.3. Data collection and outcome assessment

Patient demographics, including age, sex, and body mass index (BMI), were recorded. We collected initial laboratory findings obtained during hospitalization, including serum creatinine and cystatin C levels.

Serum creatinine and cystatin C levels were measured at the hospital laboratory. Serum creatinine levels were measured using the enzymatic method (Beckman Coulter, Japan), while serum cystatin C levels were measured using the latex agglutination turbidimetric immunoassay (Wan Tai Drd, Beijing, China). The Cre/Cys C ratio was calculated by dividing the serum creatinine value by the serum cystatin C value.

The primary outcomes included respiratory support, complications during hospitalization, hospital length of stay (LOS), admission to the intensive care unit (ICU), and mortality. Respiratory support refers to the use of noninvasive ventilators or invasive ventilators during hospitalization due to the condition. Complications are the combination of acute hypoxemic respiratory failure or acute hypercapnic respiratory failure during hospitalization. LOS was obtained by calculating the patient’s admission time and discharge time. Mortality refers to death during hospitalization at this visit.

### 2.4. Statistical analysis

SPSS 26.0 (IBM Corp, Armonk, NY) was used to analyze the results statistically. Continuous variables were first evaluated for normal distribution. The normally distributed variables are presented as the means ± standard deviations. Categorical variables are presented as frequency counts and percentages. The variables between 2 groups were compared using the independent samples *t* test or Mann–Whitney *U* test. Categorical variables were analyzed using the χ2 test. Univariable correlations between variables were assessed based on Pearson correlation coefficients. Binary logistic regression analysis was used to analyze the association between the Cre/Cys C ratio and AECOPD risk. Two models were constructed for the regression analysis: model 1 was unadjusted, while model 2 was adjusted for confounding variables, including age, sex, weight, BMI, and chronic diseases (hypertension, diabetes, and coronary heart disease). We adjusted these variables to determine whether the factors of interest were related to AECOPD risk. *P* value < .05 was defined as the significance threshold.

## 3. Results

### 3.1. Study participant characteristics

A total of 411 patients were included in the present study. The mean age of the participants was 70.49 years, and the age range was 38 to 95 years. There were 246 males (59.85%) and 165 females (40.15%). The mean Cre/Cys C ratio was 75.95 ± 14.58. The noninvasive ventilation rate, acute hypercapnic respiratory failure rate, ICU admission rate, and mortality rate were 11.68%, 22.87%, 2.92%, and 0.24%, respectively.

In the low Cre/Cys C ratio group, there were no significant differences in the different airflow limitation severity groups, including weight (*P* = .113), BMI (*P* = .445), creatinine level (*P* = .419), cystatin C level (*P* = .875), and Cre/Cys C ratio (*P* = .255). However, the age of the mild group was significantly higher than that of the other group (*P* = .015). The male ratio was also higher than that in the other group (*P* = .000). (Table 1).

**Table 1:** Baseline characteristics of the participants according to Cre/Cys C ratios.

	Low Cre/Cys C ratio group (n = 206)						High Cre/Cys C ratio group (n = 205)						F/X^2^ value	*P* value
	Mild (n = 18)	Moderate (n = 76)	Severe (n = 67)	Very Severe (n = 45)	F/X2 value	*P* value	Mild (n = 23)	Moderate (n = 92)	Severe (n = 56)	Very Severe (n = 34)	F/X2 value	*P* value		
Age, yr, mean (SD)	77.00 ± 8.71	73.14 ± 9.92	71.67 ± 8.45	68.91 ± 10.89	3.566	.015	73.26 ± 10.86	67.30 ± 11.35	68.79 ± 8.96	70.44 ± 10.23	2.274	.081	1.207	.273
Male, n (%)	2 (11.1)	27 (35.5)	32 (47.8)	29 (64.4)	18.155	.000	18 (78.3)	70 (76.1)	40 (71.4)	28 (82.4)	1.462	.691	44.911	.000
Weight	46.89 ± 8.70	53.08 ± 10.46	50.82 ± 10.71	53.67 ± 14.23	2.012	.113	55.35 ± 9.48	58.65 ± 9.93	56.43 ± 9.36	55.24 ± 9.20	1.573	.197	6.479	.011
BMI	22.62 ± 3.03	22.85 ± 3.79	21.87 ± 4.30	21.77 ± 5.37	0.895	.445	22.56 ± 2.86	23.31 ± 3.56	22.35 ± 3.09	21.47 ± 3.34	2.796	.041	5.145	.024
FEV1/FVC	62.99 ± 4.49	58.51 ± 6.82	48.40 ± 7.39	44.65 ± 8.90	53.185	.000	64.38 ± 3.66	59.95 ± 6.86	48.01 ± 6.36	42.35 ± 7.22	97.111	.000	1.047	.307
FEV1 (% of predicted value)	100.53 ± 22.44	63.32 ± 8.94	38.14 ± 5.75	22.50 ± 4.10	396.019	.000	91.13 ± 10.48	63.14 ± 8.66	39.47 ± 5.63	23.12 ± 4.18	483.836	.000	1.087	.298
Creatinine (µmol/L)	67.21 ± 13.80	68.83 ± 17.70	68.11 ± 18.62	63.49 ± 16.88	0.947	.419	87.84 ± 37.10	87.73 ± 38.45	78.66 ± 18.96	87.93 ± 36.60	0.993	.397	15.967	.000
Cystatin C (mg/L)	1.05 ± 0.24	1.07 ± 0.29	1.05 ± 0.35	1.02 ± 0.26	0.230	.875	0.97 ± 0.31	0.99 ± 0.40	0.92 ± 0.22	1.03 ± 0.38	0.856	.465	0.773	.380
Cre/Cys C ratio	64.39 ± 7.10	65.04 ± 7.52	65.51 ± 6.51	62.74 ± 8.64	1.363	.255	89.17 ± 13.99	88.23 ± 10.64	86.20 ± 9.99	85.48 ± 8.61	1.012	.388	11.509	.001

BMI = body mass index, Cre/Cys C ratio = creatinine to-cystatin C ratio, FEV1 = forced expiratory volume during 1 second, FVC = forced vital capacity.

In the high Cre/Cys C ratio group, the moderate group had a significantly elevated BMI compared with the mild, severe, and very severe groups (*P* = .041). There were no significant differences in other parameters. The specific results are presented in Table 1.

Comparisons of parameters between the low Cre/Cys C ratio group and the high Cre/Cys C ratio group showed that the high Cre/Cys C ratio group had a significantly higher male ratio (76.1 vs 43.7%, *P* = .000), weight (57.11 ± 9.65 vs 51.93 ± 11.42, *P* = .011), BMI (22.66 ± 3.37 vs 22.27 ± 4.29, *P* = .024). There were no significant differences in other parameters. The specific results are presented in Table 1.

### 3.2. Outcomes

In the low Cre/Cys C ratio group, the noninvasive ventilation ratio (0, 1.3%, 11.9% vs 44.4%, *P* = .000) and acute hypercapnic respiratory failure ratio (11.1%, 5.3%, 28.4% vs 71.1%, *P* = .000) of the very severe group were significantly higher than those in the other group.

In the high Cre/Cys C ratio group, compared with the mild, moderate, and severe groups, the very severe group had a significantly elevated noninvasive ventilation ratio (0, 2.2%, 7.1% vs 38.2%, *P* = .000) and acute hypercapnic respiratory failure ratio (0, 5.4%, 21.4%, vs 58.8%, *P* = .000). Furthermore, the hospital LOS of the very severe group was significantly higher than that of the other group (9.04 ± 1.97, 9.59 ± 2.41, 10.45 ± 3.51 vs 11.47 ± 4.13, *P* = .005). There were no significant differences in other parameters. The specific results are presented in Table 2.

**Table 2:** Differences in the distributions of outcomes between patients with a low Cre/Cys C ratio and a high Cre/Cys C ratio.

Outcomes	Low Cre/Cys C ratio group						High Cre/Cys C ratio group						F/X^2^ value	*P* value
	Mild	Moderate	Severe	Very Severe	F/X2 value	*P* value	Mild	Moderate	Severe	Very Severe	F/X2 value	*P* value		
Respiratory support														
Non-invasive ventilation	0 (0)	1 (1.3)	8 (11.9)	20 (44.4)	47.742	.000	0 (0)	2 (2.2)	4 (7.1)	13 (38.2)	30.298	.000	2.304	.129
Invasive ventilation	0	1 (1.3)	0	0	2.574	1.000	0	0	0	0			-	1.000
Complications														
Acute hypoxemic respiratory failure	1 (5.6)	5 (6.6)	7 (10.4)	1 (2.2)	2.946	.400	1 (4.3)	9 (9.8)	7 (12.5)	3 (8.8)	1.055	.773	1.186	.276
Acute hypercapnic respiratory failure	2 (11.1)	4 (5.3)	19 (28.4)	32 (71.1)	63.979	.000	0	5 (5.4)	12 (21.4)	20 (58.8)	53.612	.000	5.392	.02
Clinical outcomes														
Hospital LOS (d)	9.33 ± 2.77	10.21 ± 6.42	11.39 ± 4.25	12.36 ± 4.03	2.498	.061	9.04 ± 1.97	9.59 ± 2.41	10.45 ± 3.51	11.47 ± 4.13	4.354	.005	5.243	.023
Admission to the ICU	0	5 (6.6)	2 (3.0)	0	4.593	.204	1 (4.3)	1 (1.1)	2 (3.6)	1 (2.9)	2.326	.461	0.333	.564
Mortality	0	1 (1.3)	0	0	2.574	1.000	0	0	0	0			0.998	.318

Cre/Cys C = ratio of creatinine to cystatin C, ICU = Intensive care unit, LOS = Length of stay in the hospital

Comparisons of parameters between the low Cre/Cys C ratio group and the high Cre/Cys C ratio group showed that the low Cre/Cys C ratio group had a significantly higher acute hypercapnic respiratory failure ratio (27.7 vs 18%, *P* = .02), hospital LOS (10.99 ± 5.09 vs 10.07 ± 3.11, *P* = .023). There were no significant differences in other parameters. The specific results are presented in Table 2.

### 3.3. Analysis of the correlation between the Cre/Cys C ratio and clinical parameters

**Figure 1.**
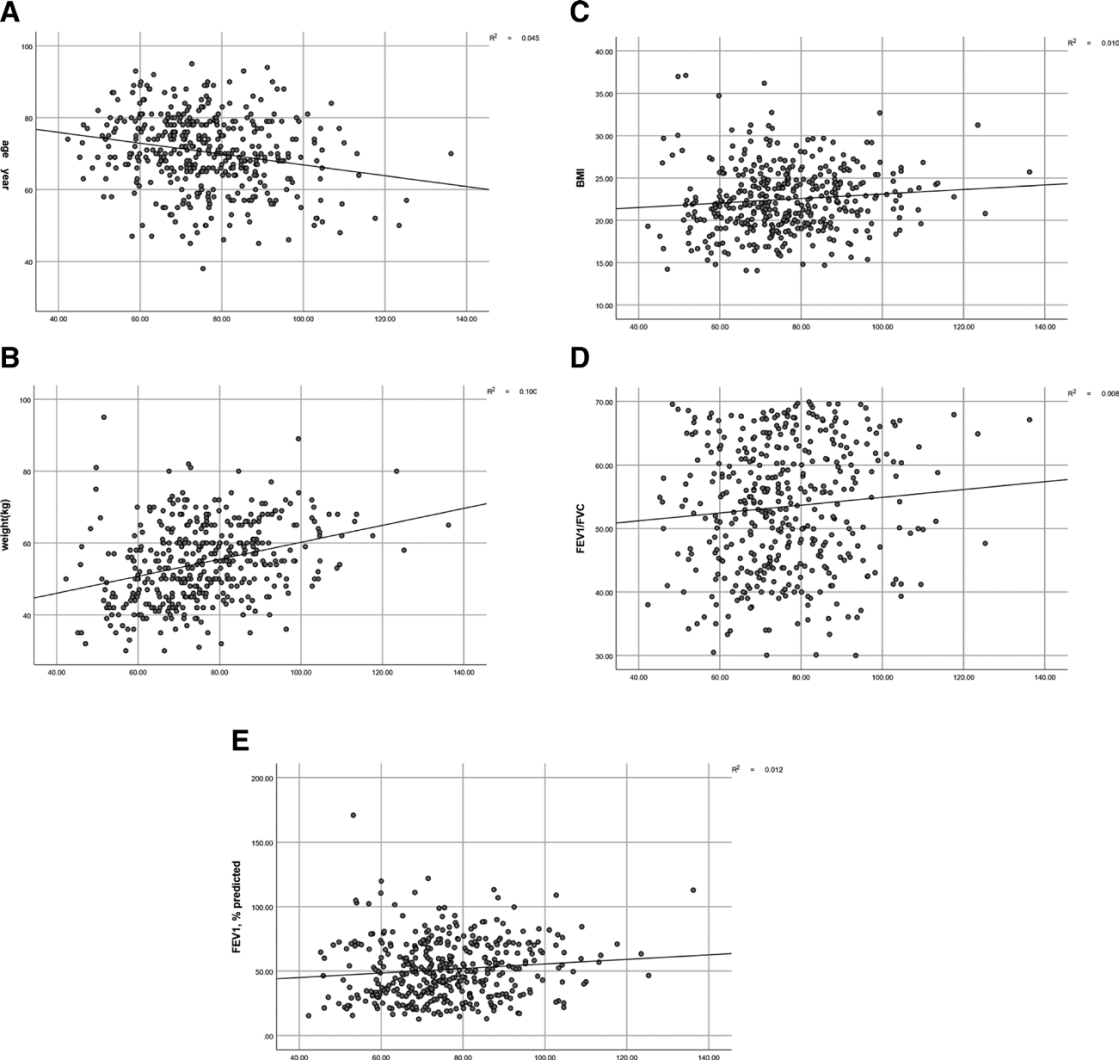
Analysis of the correlations between the serum Cre/Cys C ratio and clinical parameters such as patient age, weight, BMI, the FEV1/FVC ratio, and predicted FEV1. BMI = body mass index, Cre/Cys C = ratio of creatinine to cystatin C, FEV1 = forced expiratory volume during 1 second, FVC = forced vital capacity.

With regard to study parameters, the Cre/Cys C ratio was significantly associated with age (r = −0.206, *P* = .000; Fig. 1A, Table 3), weight (*R* = 0.331, *P* = .000; Fig. 1B, Table 3), BMI (*R* = 0.133, *P* = .007; Fig. 1C, Table 3), FEV1/FVC ratio(*R* = 0.088,*P* = .074; Fig. 1D, Table 3) and predicted FVC% (*R* = 0.130, *P* = .009; Fig. 1E, Table 3). Finally, a multiple regression was run to predict the Cre/Cys C ratio from age, weight, BMI, FEV1/FVC ratio, and FVC% predicted. These variables significantly predicted FABP-4, with F (5, 405) = 24.571, *P* = .000, R2 = 0.233. The results showed that the most significant predictors of the Cre/Cys C ratio were age (*P* = .007), weight (*P* = .000), BMI (*P* = .000), and predicted FEV1 (*P* = .000) (Table 4).

**Table 3:** Results of the correlations between the serum Cre/Cys C ratio and clinical parameters such as patient age, weight, BMI, the FEV1/FVC ratio, and predicted FEV1.

Parameters	r	*P* value
Age (yr)	−0.206	.000
Weight (kg)	0.331	.000
BMI	0.133	.007
FEV1/FVC ratio	0.088	.074
FEV1 (% of predicted value)	0.130	.009

BMI = body mass index, Cre/Cys C = ratio of creatinine to cystatin C, FEV1 = forced expiratory volume during 1 second, FVC = forced vital capacity.

**Table 4:** Results of the multiple stepwise linear regression analysis of the factors associated with the Cre/Cys C ratio.

	95% CI	Beta	*P* value
Age (yr)	−0.302 to −0.047	−0.124	.007
Weight (kg)	0.806 to 1.238	0.762	.000
BMI	−2.733 to −1.526	−0.564	.000
FEV1/FVC ratio	−0.281 to −0.091	−0.065	.317
FEV1 (% of predicted value)	0.074 to 0.237	0.245	.000

BMI = body mass index, CI = confidence interval, Cre/Cys C = ratio of creatinine to cystatin C, FEV1 = forced expiratory volume during 1 second, FVC = forced vital capacity.

Multivariate analysis was performed to determine whether the Cre/Cys C ratio was a predictor of AECOPD risk. Model 1 showed that a low Cre/Cys C ratio was associated with an increased hospital LOS (odds ratio: −0.114, 95% confidence interval: −0.061 to −0.005) and admission to the ICU (odds ratio: 0.951, 95% confidence interval: 0.907–0.996). After adjustment for potential confounding factors, model 2 showed that a low Cre/Cys C ratio was not independently associated with AECOPD risk (Table 5).

**Table 5:** The multiple linear regression analysis results to determine the independent predictors of AECOPD risk.

Risk	Model 1			Model 2		
	95% CI	OR	*P* value	95% CI	OR	*P* value
Respiratory support non-invasive ventilation						
Low Cre/Cys C	0.921 to 1.035	0.977	.424	0.930 to 1.065	0.995	.889
High Cre/Cys C	0.956 to 1.034	0.994	.772	0.950 to 1.037	0.992	.730
Total	0.983 to 1.024	1.003	.762	0.985 to 1.035	1.010	.433
Invasive ventilation						
Low Cre/Cys C			NA			NA
High Cre/Cys C			NA			NA
Total			NA			NA
Complications acute hypoxemia respiratory failure						
Low Cre/Cys C	0.932 to 1.068	0.997	.942	0.909 to 1.054	0.979	.569
High Cre/Cys C	0.950 to 1.044	0.996	.871	0.923 to 1.023	0.972	.273
Total	0.978 to 1.026	1.002	.858	0.959 to 1.014	0.986	.327
Acute hypercapnia respiratory failure						
Low Cre/Cys C	0.949 to 1.033	0.990	.637	0.951 to 1.043	0.996	.866
High Cre/Cys C	0.976 to 1.037	1.006	.707	0.980 to 1.052	1.015	.398
Total	0.982 to 1.013	0.997	.743	0.984 to 1.022	1.003	.757
Clinical outcomes hospital LOS (d)						
Low Cre/Cys C	−0.127 to 0.061	−0.048	.492	−0.138 to 0.052	−0.063	.369
High Cre/Cys C						
Total	−0.061 to −0.005	−0.114	.021	−0.061 to 0.002	−0.101	.068
Admission to the ICU						
Low Cre/Cys C	0.877 to 1.034	0.952	.245	0.829 to 1.019	0.919	.108
High Cre/Cys C	0.820 to 1.103	0.951	.508	0.819 to 1.160	0.975	.771
Total	0.907 to 0.996	0.951	.034	0.898 to 1.011	0.953	.110
Mortality						
Low Cre/Cys C	0.605 to 1.279	0.879	.501			NA
High Cre/Cys C			NA			NA
Total	0.899 to 1.154	1.018	.775			NA

Model 1: non adjusted model.

Model 2: adjusted for age, sex, weight, BMI, FEV1/FVC ratio, predicted FEV1, and the presence of chronic diseases (hypertension, diabetes, and coronary heart disease).

AECOPD = acute exacerbation of chronic obstructive pulmonary disease, CI = confidence interval, Cre/Cys C = ratio of creatinine to cystatin C, ICU = intensive care unit, LOS = hospital length of stay, NA = unable to analyze due to too few events, OR = odds ratio.

## 4. Discussion

The Cre/Cys C has previously been used as a surrogate marker of muscle mass in amyotrophic lateral sclerosis,^[7]^ in patients undergoing physical checkups,^[14]^ in ICU patients,^[15]^ in lung transplant candidates,^[16]^ and in type 2 diabetic patients.^[17]^ To the best of our knowledge, based on the available literature, this is the first study to explore the corporation of the Cre/Cys C ratio, a rapidly measured and widely available biomarker, with risk in patients with AECOPD.

Several previous reports showed that the Cre/Cys C ratio was negatively correlated with age,^[8,18–20]^ sex,^[19]^ BMI,^[8,19,20]^ and waist circumference.^[20]^ In addition, there were positive correlations between the Cr/Cys C ratio and FEV1,^[18,21]^ FVC,^[21]^ and FVC% predicted.^[8]^ Our results also showed that the male ratio, weight, and BMI levels were significantly elevated with a high Cre/Cys C ratio in patients with acute exacerbation of COPD. There was a negative correlation between the Cre/Cys C ratio and age. We consider the reason that it has been reported that muscle mass decreases 1% to 2% annually in patients with COPD over 50 years of age,^[22]^ but 5% to 13% of patients over 65 years of age without chronic diseases such as COPD develop sarcopenia.^[23]^ On the other hand, there was a positive correlation between the Cre/Cys C ratio and weight, BMI, and FVC% predicted. Except for BMI, the findings were similar to those of previous studies. However, a prospective study consecutively recruited patients aged 60 years and older and found that the Cre/Cys C ratio was positively correlated with BMI.^[24]^ Previous reports that the Cre/Cys C ratio is a marker for muscle-adjusted visceral fat mass^[20]^ could explain this finding.

A number of studies have explored the risk factors associated with mortality in patients with AECOPD thus far, and it is known that albumin, respiratory rate, blood gas analysis (PCO2, hemoglobin, lactic acid, etc), inflammation-related indicators, etc, are important prognostic factors for mortality in these patients.^[13,25–29]^ Our study used another easy and inexpensive tool to assess the risk of AECOPD.

The serum Cre/Cys C ratio has been reported as a predictive marker for the adverse effects of chemotherapy in lung cancer. In recent studies, the association of the Cre/Cys C ratio with clinical outcomes (e.g., malnutrition, frailty and hospital stay),^[24]^ hospital admission,^[21]^ 3-year all-cause mortality,^[24]^ cardiovascular disease,^[9]^ risk of pneumonia,^[19]^ and predictors of severe exacerbations 1 has been reported. Our results indicated that the acute hypercapnic respiratory failure ratio and hospital LOS were significantly higher in patients with a low Cre/Cys C ratio than in those with a high Cre/Cys C ratio. The low Cre/Cys C ratio showed a significant association with risk in patients with AECOPD, including increased hospital LOS and admission to the ICU ratio. There was no independent association with AECOPD risk following multivariable adjustments. A low Cre/Cys C ratio in patients with COPD is considered to be associated with a high risk of hospitalization, as systemic inflammation due to acute exacerbations causes muscle weakness and poor health.^[30]^

Our study had some limitations. Previous studies have shown that a higher Cre/Cys C ratio was independently associated with a lower risk of 3-year all-cause mortality after adjusting for potential confounders. The mortality in our study was low and we were unable to determine the correlation between mortality and the Cre/Cys C ratio. Furthermore, it must be replicated in multicentric studies, using a higher number of patients coming from different areas. Finally, this study did not consider the long-term prognosis of the patients, and the prognosis of the patients after discharge could be followed up with in future studies to better assess the long-term risk of patients with AECOPD.

## 5. Conclusion

In conclusion, the present study indicated that the Cre/Cys C ratio is an easy, cheap, repeatable, and promising tool that allows us to evaluate the risk of AECOPD using serum markers. Additionally, a low Cre/Cys C ratio may be a valuable predictor of the risk of AECOPD. A low Cre/Cys C ratio was associated with a prolonged LOS and admission to the ICU in AECOPD patients. However, the associations were not independent.

## Author contributions

**Data curation:** Liang He, Yan Li, Xijun Gou.

**Formal analysis:** Liang He.

**Writing – original draft:** ling lei.

**Writing – review & editing:** ling lei.
